# The Neural Substrate of Positive Bias in Spontaneous Emotional Processing

**DOI:** 10.1371/journal.pone.0015454

**Published:** 2010-11-08

**Authors:** Roberto Viviani, Hanna Lo, Eun-Jin Sim, Petra Beschoner, Julia C. Stingl, Andrea B. Horn

**Affiliations:** 1 Human Brain Mapping Unit, Department of Psychiatry, University of Cambridge, Cambridge, United Kingdom; 2 Department of Psychiatry and Psychotherapy III, University of Ulm, Ulm, Germany; 3 Psychotherapy and Psychosomatic Clinic, University of Ulm, Ulm, Germany; 4 Department of Clinical Pharmacology, University of Ulm, Ulm, Germany; 5 Division of Psychopathology and Clinical Intervention, Department of Psychology, University of Zurich, Zurich, Switzerland; The University of Melbourne, Australia

## Abstract

Even in the presence of negative information, healthy human beings display an optimistic tendency when thinking of past success and future chances, giving a positive bias to everyday's cognition. The tendency to actively select positive thoughts suggests the existence of a mechanism to exclude negative content, raising the issue of its dependence on mechanisms like those of effortful control. Using perfusion imaging, we examined how brain activations differed according to whether participants were left to prefer positive thoughts spontaneously, or followed an explicit instruction to the same effect, finding a widespread dissociation of brain perfusion patterns. Under spontaneous processing of emotional material, recruitment of areas associated with effortful attention, such as the dorsolateral prefrontal cortex, was reduced relative to instructed avoidance of negative material (*F*_1,58_ = 26.24, *p* = 0.047, corrected). Under spontaneous avoidance perfusion increments were observed in several areas that were deactivated by the task, including the perigenual medial prefrontal cortex. Furthermore, individual differences in executive capacity were not associated with positive bias. These findings suggest that spontaneous positive cognitive emotion regulation in health may result from processes that, while actively suppressing emotionally salient information, differ from those associated with effortful and directed control.

## Introduction

An aspect of healthy mental functioning is the positive connotation of thoughts arising spontaneously or occasioned by external stimuli, as is demonstrated by the reversal of this positive bias in depression [1] and the protective effect of optimism for mental and physical health [2]. Understanding the nature of this tendency is an important task for shedding light on the processes at the basis of mood maintenance and disorders of affect.

Because it is active even if negative stimuli are present [3], the tendency to select positive thoughts suggests the existence of a regulatory mechanism to exclude negative content. There are clear instances in which avoiding negative cognitions follows an intentional, effortful strategy, as when one tries to put worries to rest [4] or explicitly reappraises stimuli in a more favourable light [5]. In these situations, effortful control and reflective reasoning are responsible for keeping negative thoughts at bay. This conclusion is consistent with attributing to cognitive control processes, like those underlying voluntary effort, a decisive role in determining the direction of thoughts and maintaining a positive mood [6]–[7], and with modelling psychiatric disorders as involving the failure of such control [8]–[11]. These accounts are based on the insight of neuroimaging studies of emotion investigating the effect of the presence of emotional information while its role as target or distracter is defined by an explicit instruction or the task. By focusing on the capacity of top-down control processes to second or resist the attentional pull of emotional stimuli, these studies have demonstrated the crucial role played in this respect by the prefrontal cortex [5], [12]–[15].

Outside the field of emotion regulation, however, examples of positive bias are known that appear to arise automatically when evaluating choices or risks [16]–[17]. Here, bias does not emerge from the use of reflective strategies, but is a spontaneous and natural response. In these situations, positive bias may be understood as an ‘affect heuristic’ based on processes that differ in kind from those of reflective reasoning [18]–[19]. While this field of inquiry has developed separately from that of the neurobiology of emotion, it potentially provides an alternative account of how positive bias may emerge in everyday life, and by implication, of how emotion regulation may be achieved without resorting to cognitive control [20]. Indeed, theorists of emotion regulation have recently turned their attention to the possible role of ‘automatic’ forms of emotion regulation [21], and emphasized the importance of mapping its possibly distinct neural substrate [22]. However, notwithstanding their theoretical importance, the empirical investigation of automatic emotion regulation has so far remained elusive.

Positive bias in cognitions is demonstrable by asking participants to assemble sentences from a set of scrambled words (*scrambled sentences task*). When legal sentences with different meanings are possible given the words in the set, a choice is implicitly made among the alternative solutions to the task. For example, “bright the very dismal looks future” may be unscrambled to form a negative thought, “the future looks very dismal,” or a positive thought, “the future looks very bright.” When the alternative sentences have an affective meaning, as in this example, healthy participants are remarkably selective and spontaneously tend to avoid the pessimistic version, forming 70–80% positive sentences even if the existence of emotional words is not mentioned in the explanation of the task [3]. Crucially, this occurs even if negative words are emotionally more salient than positive words, showing that a mechanism must actively steer thoughts towards optimism. If the spontaneous responses in this task were passively determined by the automatic attention-grabbing emotional salience of the input [23]–[24], participants would form more negative than positive sentences, not less. In depression research, where the scrambled sentences task is established, is has been shown that in contrast to the positive bias of health participants individuals suffering from major depression show a pronounced negative bias and form by far more negative sentences [25]–[26].

When applied to the scrambled sentences task, the effortful control and the affect heuristic views provide alternative accounts of the avoidance of negative sentences in healthy individuals, corresponding to the two fundamentally different hypotheses on how emotion may be integrated into the cognitive control architecture. In the effortful control account, emotional information in the presented words is first flagged as relevant by processes appraising emotional material in the input channel, which subsequently trigger the intervention of executive attentional processes configured so as to intentionally exclude negative material in the chosen response [4]–[5], [27]–[28]. Under the affective heuristic hypothesis, in contrast, the spontaneous preference for positive sentences depends on processes determining response that are automatic in the sense of being active regardless of the subject's intentions, and do not require effort or the supervision of executive attention [20], [29]–[31]. Hence, the scrambled sentences task offers the opportunity to empirically investigate the nature of mechanisms steering thoughts and emotional response spontaneously and test the differing predictions of the effortful and affect heuristic models.

We undertook a direct comparison of spontaneous and intentional avoidance of negative content in the scrambled sentences task using a neuroimaging technique that allows quantitative estimation of perfusion levels (continuous arterial spin labelling, CASL [32]–[33]). After a resting baseline of 8 min, participants were asked to unscramble sentences in homogeneous task blocks. The sentences to be formed belonged in two conditions: *neutral* (no emotional content in the possible sentences), and *emotional* (in each trial, the alternative formed sentences could be positive or negative). Negative words were significantly more arousing than positive words (see Methods for details). Furthermore, the instruction varied across participants. A first group was simply told to form grammatically correct sentences, eliciting the spontaneous avoidance of the negative alternative (*spontaneous group*); a second group, in contrast, was explicitly instructed to avoid forming negative sentences (*instructed group*). Both groups formed sentences under the neutral control and affective condition. We reasoned that, if the effortful control account is correct, a recruitment of brain areas associated with executive processes must be observed in both the spontaneous and instructed groups when confronted with emotional ambiguous material, since avoiding negative sentences would place similar increased demands on the executive attentional network relative to the neutral condition, where sentence formation is unconstrained [34]. By contrast, the affective heuristic account predicts dissimilar perfusion patterns in the spontaneous and instructed groups, in accordance with the expected different nature of processes involved in effortful and spontaneous response elaboration. Furthermore, if the natural heuristic account is correct, areas specifically activated by the exposure to emotional material in the spontaneous group should provide information about the brain networks associated with spontaneous avoidance of negative content.

To characterize further the nature of avoidance of negative sentences, we measured the working memory capacity (WMC) of participants using a complex memory span test (OSPAN [35]). Working memory capacity is the psychometric notion that comes closest to the concept of central executive [36]. Increased success in voluntary avoidance of negative content in individuals with higher working memory capacity has been shown in a previous study of instructed avoidance [37]. We reasoned that, if the effortful control hypothesis is correct, participants are expected to rely on executive processes to exclude negative material even without explicit instructions. In this case, individual differences in working memory capacity must have an effect on the degree of success in avoiding negative outcomes even in the spontaneous condition. No such effect is predicted by the affective heuristic model, because cognitive capacity is not associated with the performance of heuristic strategies [38]–[39]. We also administered the scrambled sentences task before scanning in the original paper and pencil form [3] to allow participants to practice the task, and reliably verify the existence of the positive bias in participants. The two groups, spontaneous and instructed, were matched for age, sex, OSPAN scores, and depressiveness.

By contrasting spontaneous and voluntary forms of avoidance of negative content, we show that areas associated with executive control are recruited less under spontaneous response. Furthermore, we provide evidence for the dissociation between the neural substrates of spontaneous and voluntary emotion regulation. We anticipate that the identification of these spontaneous mechanisms of emotion regulation will improve our understanding of psychiatric disorders of affect.

## Results

### Behavioural Data

As anticipated, participants avoided negative sentences in the spontaneous group (25% negative sentences, logistic regression *z* = −11, *p*<0.001) as well as in the instructed group (0.5% negative sentences). In the spontaneous group, depressiveness scores were associated with rates of negative sentences (*t* = 3.17, *p* = 0.004), even if these scores were all low and in the normal range. In contrast with the prediction of the effortful control hypothesis, the association between WMC and avoidance of negative sentences in the spontaneous group was not significant (logistic regression, *z* = −1.2, *p* = 0.22). However, WMC scores did predict the proportion of well-formed sentences (negative or positive) formed in the task (*z* = 3.8, *p*<0.001), both in the spontaneous (*z* = 3.5) and in the instructed group (*z* = 2.7). Rates of negative sentences formed during scanning and in the pre-scan test were highly correlated across subjects (Spearman's rank correlation *r* = 0.48, *p*<0.001).

### Neuroimaging Results

**Figure 1:**
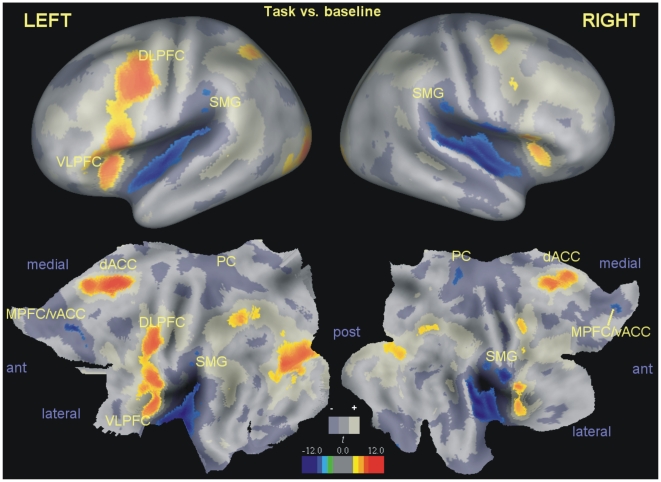
Main task perfusion changes. Changes in brain perfusion in the sentences building task, irrespective of emotional valence or instruction, overlaid by ‘fiducial mapping’ on an atlas of cortical surface based on 12 individuals (top row) and on the corresponding flat map (bottom row). Voxel-level corrected activations relative to the resting baseline are displayed in yellow/orange, deactivations in blue/green (*p*≤0.05). In shades of gray are shown the activations and deactivations (+/−) at uncorrected significance levels (*p*≤0.05). DLPFC: dorsolateral prefrontal cortex; VLPFC: ventrolateral prefrontal cortex; SMG: supramarginal gyrus; MPFC/vACC: medial prefrontal cortex (pregenual) and ventral anterior cingulate cortex; dACC: dorsal anterior cingulate; PC: posterior cingulate; ant, post: anterior, posterior directions.

To verify that the perfusion technique was successful, and identify areas involved in the task, we first contrasted the conditions in which subjects were forming sentences, irrespective of valence (emotional or neutral) and instruction (explicit instruction or spontaneous avoidance of negative sentences), against the baseline perfusion at rest. This preliminary contrast revealed the activation of areas known to be involved in attentional and linguistic processing [40], such as posterior parietal, dorsal cingulate, the dorsolateral prefrontal cortex (DLPFC) extending superiorly towards the precentral sulcus (frontal eye fields, FEFs), and the left ventrolateral prefrontal cortex/anterior insula (VLPFC/AI). Also deactivations relative to the baseline were found that replicated well-known observations in the field comparing the default mode with the initiation of cognitive processes [41]–[48], and involved the posterior and ventral cingulate, posterior insula and the adjacent parietal operculum and superior temporal gyrus, and a band extending from the inferior parietal posteriorly to the inferior temporal gyrus anteriorly (Figure 1 and Supporting Table S1 in the information available online).

**Figure 2:**
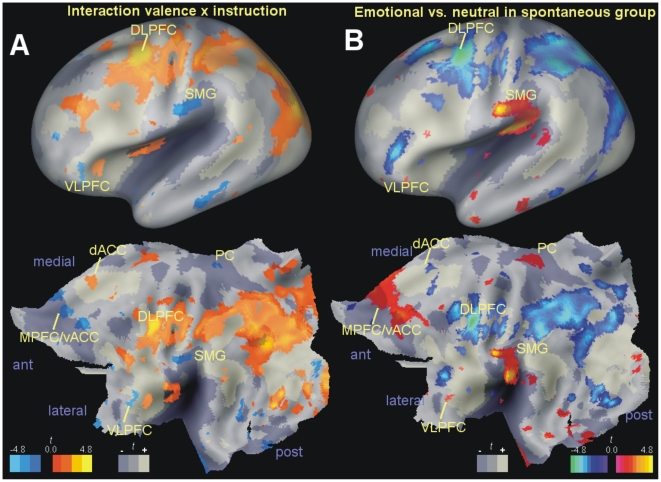
Neuroimaging of controlled and spontaneous avoidance of negative content. ‘Fiducial mapping’ of contrast effects on the inflated cortical surface (top row) and on the corresponding flat map (bottom row) for the left hemisphere is shown. In both panels, shades of gray indicate the same areas activated or deactivated by the task at uncorrected significance levels (as detected by a comparison with the baseline condition) as in Figure 1. A: cortical projection rendering of the emotional valence×instruction interaction. In red/yellow are areas following the pattern in DLPFC/FEF indicating more perfusion in the instructed relative to the spontaneous group when processing emotional information. In blue are areas in which the interaction went in the opposite direction. B: fiducial mapping of the contrast emotional vs. neutral in the spontaneous group only. The largest areas of increased perfusion during exposure to emotional words were located in or near areas deactivated by the main task (MPFC/vACC, PC, SMG). To be as inclusive as possible (see text for rationale), statistical maps were thresholded at *p*≤0.05, uncorrected. Abbreviations as in Figure 1.

**Figure 3:**
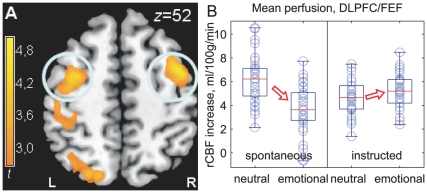
Voluntary and spontaneous regulation in the dorsal network. A: Displayed perfusion values are bilateral averages of the signal in the DLPFC/FEF cluster in BA6. To avoid selection bias, the cluster was defined from the contrast sentence formation vs. baseline, irrespective of valence or instruction, not from the interaction contrast, using the threshold *p*≤0.05, voxel-level corrected. B: Mean blood flow increases relative to baseline in the spontaneous (left half) and the instructed condition (right half). Spontaneous avoidance, triggered by the presence of emotional words, is associated with less recruitment of this control area. Under instructed avoidance, in contrast, perfusion increased (interaction: *F*_1,57_ = 11.59, *p* = 0.001). This increase was small, but participants were seconding a natural tendency, not opposing a prepotent response.

To test for the dissociation predicted by the affect heuristic hypothesis, we looked for an interaction between sentence condition (emotional vs. neutral) and instruction (explicit instruction vs. spontaneous avoidance of negative sentences), using the areas of task activation and deactivation as an interpretive guide (Figure 2A and Supporting Table S2 in the online information). This interaction was significant in the most dorsal portion of the DLPFC cluster activated by the task (Figure 2A), reaching its peak in the right FEF, Brodmann area 6 (BA6, *x*, *y*, *z*: 44, 4, 52, *F*_1,58_ = 26.24, *p* = 0.047, corrected at voxel level for the whole volume). Further analysis revealed that the interaction was due to reduced perfusion in the emotional relative to the neutral condition in spontaneous participants (*x*, *y*, *z*: 44, 4, 52, *t* = −5.67, *p* = 0.030, corrected), while in instructed participants perfusion increased slightly (*x*, *y*, *z*: 48, 0, 52; *t* = 2.23, *p* = 0.017; see Figure 3). In the left DLPFC/FEF, a similar pattern was observed, even if it failed to reach significance at corrected levels (interaction, *x*, *y*, *z*: −34, −4, 54, *F*_1,58_ = 19.05, *p*<0.001; simple effect in spontaneous participants, *x*, *y*, *z*: −36, −2, 54, *t* = −4.55, *p*<0.001; in instructed participants, *x*, *y*, *z*: −40, −12, 54, *t* = 2.65, *p* = 0.007, all significance levels uncorrected). This same interaction extended posteriorly on the left to the superior parietal areas activated by the task (Figure 3).

**Figure 4:**
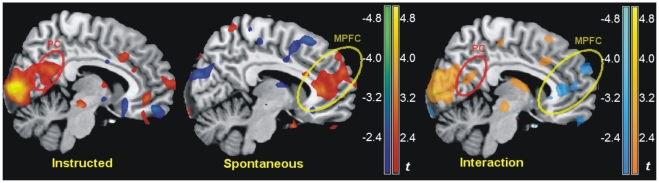
Voluntary and spontaneous regulation in the medial wall. Contrast emotional vs. neutral in the instructed and spontaneous group separately (left), and in the interaction (right) in the medial wall (*x* = −7 mm), overlaid on a standard template brain. In the interaction, in light blue are increases of perfusion due to the presence of emotional words in the spontaneous group relative to the instructed group. PC: precuneus/posterior cingulate; MPFC: perigenual medial prefrontal cortex. Maps of *t* values were thresholded for illustration purposes at *p*≤0.05, uncorrected.

To further investigate the mechanism of spontaneous control we undertook an exploratory analysis of changes in perfusion in the spontaneous group, contrasting exposure to emotional with neutral material, and using the areas of task activation and deactivation detected by perfusion imaging to aid interpretation (Figure 2B and Supporting Table S3 online). This contrast revealed perfusion increases (red/yellow), involving the superior temporal and the supramarginal gyurs (SMG, *x*, *y*, *z*: −64, −10, 28, BA43, *t* = 4.73, *p*<0.001), the perigenual medial prefrontal cortex (perigenual MPFC, *x*, *y*, *z*: −8, 38, 6, *t* = 3.20, *p* = 0.002, Figure 4), and the inferior temporal gyrus (ITG, *x*, *y*, *z*: −54, −22, −26, BA 20, *t* = 3.08, *p* = 0.002, all significance levels uncorrected). These activations were more marked in the spontaneous than in the instructed group, as these same areas appear in the interaction between groups and condition in Figures 2A (in yellow) and 4 (perigenual MPFC, *x*, *y*, *z*: −8, 38, 6, *F*_1,58_ = 9.05 [*t* = −3.01], *p* = 0.004; insula/prefrontal operculum, *x*, *y*, *z*: −64, −20, 30, *F*_1,58_ = 10.20 [*t* = −3.19], *p* = 0.002; ITG, *x*, *y*, *z*: −50, −24, −22, *F*_1,58_ = 8.23 [*t* = −2.87], *p* = 0.006, all uncorrected). Furthermore, these activations were prevalently localized to areas that were deactivated by the main task (to be as inclusive as possible in defining these areas, and avoid missing small task activations, in Figure 2 the display threshold was kept low). The deactivation was significant in the superior temporal/supramarginal gyrus (*x*, *y*, *z*: −58, −30, 20, *t* = −5.47, *p* = 0.01, corrected for the whole volume), and present as a trend in the perigenual MPFC (*x*, *y*, *z*: 0, 30, 6, *t* = −3.98, *p*<0.001, uncorrected).

**Figure 5:**
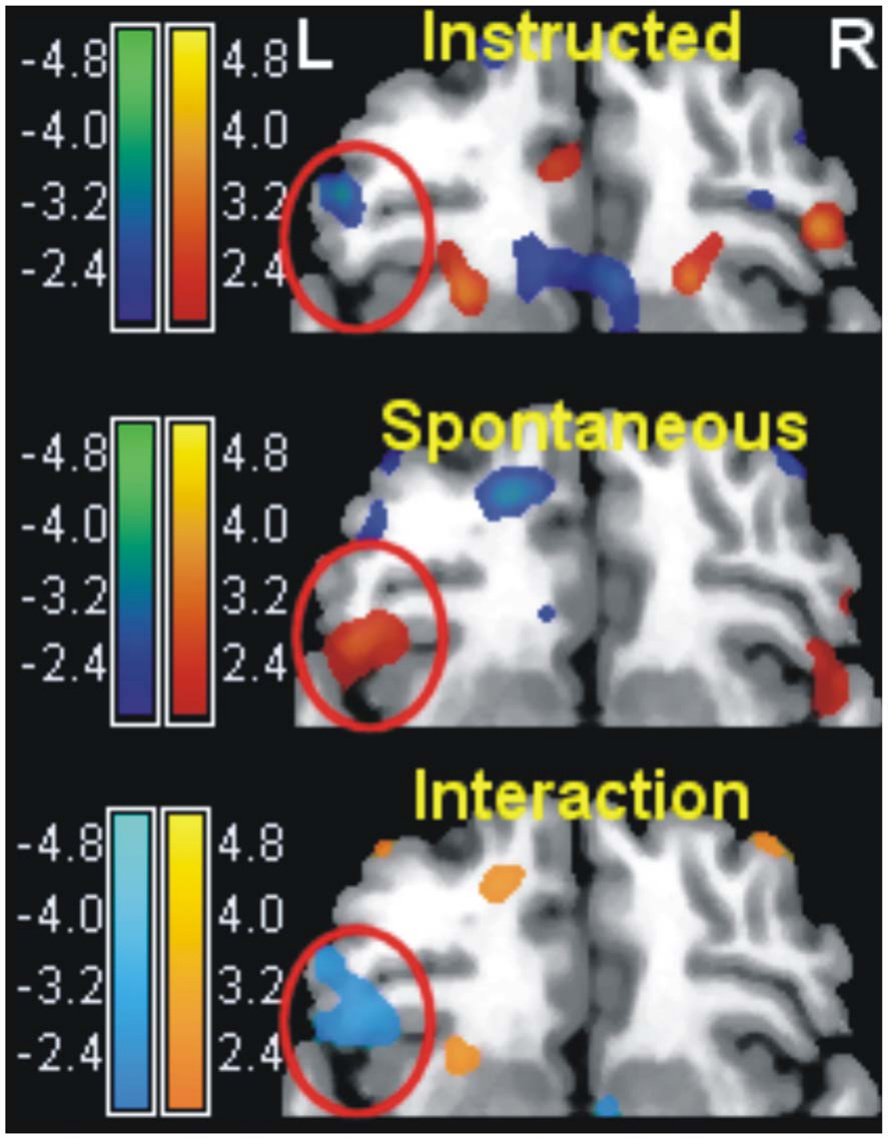
Voluntary and spontaneous regulation in the ventrolateral prefrontal cortex (VLPFC). Contrast emotional vs. neutral in the instructed and spontaneous group separately (top and middle), and in the interaction (bottom) in a transversal slice across the prefrontal cortex (*z* = −10 mm), overlaid on a standard template brain. The red circle is centered on the left VLPFC. Maps of *t* values were thresholded for illustration purposes at *p*≤0.05, uncorrected.

The left ventrolateral prefrontal cortex (VLPFC) was an exception to this pattern (Figure 5). This area and parts of the adjacent anterior insula were both more active in the task as whole (*x*, *y*, *z*: −48, 22, −10, *t* = 5.37, *p* = 0.01, corrected for the whole volume), and when the spontaneous group was exposed to emotional material (*x*, *y*, *z*: −56, 28, 22, *t* = 3.08, *p* = 0.002; *x*, *y*, *z*: −48, 22, −10, *t* = 2.59, *p* = 0.006, all uncorrected). Analysis of the interaction of these areas indicated that these small activations were slightly larger in the spontaneous group (*x*, *y*, *z*: −55, 28, 23, *F*_1,58_ = 4.36 [*t* = −2.09], *p* = 0.04, uncorrected; *x*, *y*, *z*: −42, 24, −10, *F*_1,58_ = 6.07 [*t* = −2.46], *p* = 0.017, uncorrected). A similar pattern was present on the right (*x*, *y*, *z*: 32, 20, −22, *t* = 2.72, *p* = 0.004, uncorrected; interaction, *F*_1,58_ = 9.98 [*t* = −3.16], *p* = 0.003, uncorrected).

Outside the dorsal attentional network, instructed participants showed increased perfusion levels in the presence of emotional material in the occipital/calcarine cortex, and, in the medial wall, the cuneus, precuneus and posterior cingulate (Figure 4 and Supporting Table S4 online).

## Discussion

Two independent sets of findings presented here support the notion that spontaneous avoidance of negative content reflected mechanisms distinct from explicit control, as predicted by the affect heuristic model of spontaneous avoidance. Firstly, when spontaneously avoiding negative sentences, there was less recruitment of executive attentional areas when exposed to emotional ambiguous material (where avoidance of negative content occurred) than to neutral material (in which no avoidance was required). In contrast, instructed avoidance increased this recruitment, most notably in DLPFC/FEF, an area involved in effortful attending to the visual environment in conformance to current goals and expectations [49]–[50]. This pattern speaks against attributing spontaneous avoidance to less systematic or intense recruitment of effortful control processes (irrespective of how erratic and weak), for in this case perfusion in areas associated with executive control would still have been slightly increased or unchanged under spontaneous avoidance, not decreased. For the same reason, this pattern cannot be explained by the much greater efficiency of instructed avoidance.

Secondly, working memory capacity had little influence on the rate of the spontaneous avoidance of negative sentences. This was not due to the failure of working memory capacity scores to account for variance in performance, since working memory capacity was associated with the number of sentences formed overall. Hence, these scores were associated with the participants' success in carrying out the task deliberately attended to, but not their success in the spontaneous avoidance of negative sentences.

Neuroimaging studies of control of emotional content have investigated the effect of the presence of emotional information while its role as target or distractor is defined by an explicit instruction [5], [12]–[15]. In the presence of an explicit instruction, however, strategies of emotional control due to spontaneous mechanisms cannot be identified. The evidence presented here that spontaneous mechanisms of regulation are distinct from those dependent on effortful control opens a new venue of inquiry about the mechanisms in which the healthy propensity for optimism is altered.

While this study was primarily designed to answer the question of whether the effortful control hypothesis adequately accounts for spontaneous forms of emotion regulation, it is natural to query the data about the pattern of changes induced by emotional material in the spontaneous group, in view of the information it may provide on how a model of spontaneous regulation may look like. While many of the changes observed in the spontaneous group may have been induced by the presence of emotional stimuli as such, a specific mechanism must have been active to influence the direction of thoughts without recurring to voluntary control. Remarkably, in the spontaneous group very little increased recruitment induced by the presence of emotional words was present in the areas activated by the task, especially compared to the extent of activation of the dorsal attentional network associated with the explicit instruction.

Two systems emerged in this study as candidate neural correlates of response modulation in spontaneous avoidance. The first was given by the piece-meal reversal of the typical pattern of activations and deactivations associated with focussed execution of a task relative to an unconstrained resting baseline [41], [51]–[52], which characterized the response to emotional material in the spontaneous group, and involved less recruitment of dorsal attentional areas. Notably, this partial reversal included the perigenual MPFC, which constitutes the ‘affective’ part of medial prefrontal cortex [53] and is often involved in tasks requiring handling emotional stimuli [12], [54]–[55], decisions based on heuristics [56], or imagining the future [57]–[58]. The subgenual MPFC, in particular, has been implicated in depression [59]–[60].

The second were the areas in VLPFC that were more recruited in the presence of emotional material also during spontaneous control, and in this respect dissociated from more dorsal areas in the prefrontal cortex. This dissociation parallels the known functional dissociation of their right-hemisphere homologues in studies of spatial attention, in which dorsal areas implement voluntary and effortful attentional process, while a ventral network is involved in attentional reorienting according to behavioural relevance and irrespective of perceptual salience [61]–[62]. As in the present study, in attentional tasks the ventral network is mostly deactivated, with the possible exception of VLPFC [61]. This parallelism lends further plausibility to a role of VLPFC in response modulation during spontaneous positive affective heuristics. Studies of the left VLPFC are consistent with this interpretation, showing its recruitment in working memory tasks even when typical executive functions such as encoding and planning are not active [63], and in semantic retrieval tasks, which may be involved in extracting emotional meaning from contextual information [64]. Interestingly, in a mediation analysis on deliberate emotion regulation, VLPFC activity mediated regulation success [15]. This also points to the hypothesis that this region has a special role in the regulation of affective reactions – seemingly in spontaneous as well as in deliberate emotion regulation.

As a whole, the modulation of activations and deactivations associated with instructed and spontaneous avoidance suggests a partial transfer of processing resources from a dorsal to a ventral network of cortical areas. The relative recruitment of ventral areas deactivated by the task during spontaneous avoidance is consistent with current models of the function of the default mode network emphasizing its role in spontaneous [58] or associative thinking [65]. This finding is also consistent with previous observations of a reciprocal interaction between dorsal and ventral networks [60], [66], and its modulation under the effect of emotional variables such as stress or anxiety [67] or depression [59]. When not instructed by or reporting to executive attentional processes, this ventral system may give rise to response sets that appear to be instantiated autonomously and be constitutive for mental health.

## Materials and Methods

### Recruitment and Behavioural Assessment

All participants were recruited through local announcements and gave written informed consent. The study was conducted at the Department of Psychiatry at the University of Ulm, Germany, in accordance with the Declaration of Helsinki and was formally approved by the Ethical Review Board of the University of Ulm. All subjects had normal or corrected-to-normal vision and were right-handed German native speakers. Ongoing medical, neurological, or psychiatric disease (including depression), and current use of psychotropic medication were excluded through an individualized interview with an experienced clinician (R.V. or P.B.). Depressiveness was assessed with a computerized German Version of the Centre for Epidemiologic Studies Depression Scale (CES-D, [68]; German Version: [69]). Participants with CES-D scores larger than 24 (the cut-off for suspicion of clinical depression) were excluded from the study. Working memory capacity was measured outside the scanner with the operation span test (OSPAN, [35], German version, [70]). There were 60 participants in the study (30 males), 30 per group, with age ranging from 19 to 45 (mean age 25.2, standard deviation 5.8). Participants were randomized to the groups after matching for sex, age, CES-D and OSPAN scores to control for the influence of these variables. Because individuals with a vulnerability to depression may use a different strategy than other individuals to exclude negative sentences [3], the match on CES-D scores was prioritized. As a result, the spontaneous and the instructed groups did not differ in sex (logistic regression, *z* = 0.06), age (*z* = 0.34, *p* = 0.73), CES-D (*z* = −0.01) or OSPAN scores (*z* = 0.31, *p* = 0.76).

### Scrambled Sentences Task

The scrambled sentences task was presented on two occasions: before the scan, to collect data on cognitive bias comparable to those of ref. [3], and during the scan. In both cases, a computer interface was used to present the stimuli (Presentation 9.20, Neurobehavioral Systems Inc., Albany, CA), but in the pre-scan version participants wrote down the sentence on a sheet of paper. After translation into German, the original 40 scrambled sentences used by ref. [71] were presented in white lowercase letters on a black computer screen in randomized order for three seconds in each trial. A cross in the middle of the screen alerted them to the appearance of the next stimulus. Participants had 12 seconds to write down a grammatically correct sentence with five out of six words, and had been instructed to work quickly, not to correct mistakes, and to complete as many sentences as possible. As in previous studies [3], [71], sentences were presented in two blocks of 2.5 minutes each. The test score was computed as the ratio of the negative to the total number of sentences formed.

In the scan version, the original task was modified in two respects. First, to avoid the artefacts possibly produced by speaking out loud or writing, participants were instructed to build the sentences in mind and press a button to confirm that a sentence had been formed. Scrambled sentences were presented through goggles masking the whole field of vision (Resonance Technology Inc., Northridge, CA). Time allowed to form sentences was 3 seconds per trial, previously determined as the 95% percentile of button presses indicating completion of sentences in a preliminary pilot study, resulting in 22 scrambled sentence presentations in each 2.5 minutes-long block. This maintained a mild time pressure on the completion of sentences, and kept participants focused on the task during the block, making the block as homogenous as possible as required by the perfusion design [72]. Second, the material used to form sentences alternated between emotional and neutral. There were four blocks in counterbalanced sequence to neutralize order effects. As a check on the execution of the task, after each block participants were presented with the possible solutions to the sentences presented during the block and asked to indicate the sentence they had built, if any. To keep the scanning procedure within time limits, only 11 of the 22 sentences per block were probed in this way. Instructed and spontaneous sentence formation was varied between subjects as indicated in the methodological discussion in ref. [30]. In the instructed group, participants were asked to form positive sentence and avoid the negative alternative in the emotional blocks. In the spontaneous group, no mention was made of the existence of the emotional blocks.

To characterize the emotional salience of the words used in the task, a preliminary study was conducted on a separate sample of 19 student volunteers, recruited similarly to participants in the study. Participants were asked to rate the words in the emotional sets for arousal, using a standard visual analogue rating scale [73] ranging from 1 to 9 (low to high arousal). One volunteer completed only one third of the list, and was excluded from analysis. The average arousal rating of all words was 6.1 (s.d. 0.55). Positive words were rated with an arousal rating of 0.21 points less than negative words (about 3.5% less). This small difference was significant when tested in a repeated-measurements model [74] with positiveness and words as fixed factors (*F*_1,999_ = 9.26, *p* = 0.002).

### MRI Acquisition and Preprocessing

Data were collected at the Department of Psychiatry at the University of Ulm, Germany. All magnetic resonance imaging (MRI) data were obtained with a 3-Tesla Magnetom Allegra (Siemens, Erlangen, Germany) MRI system equipped with a head volume coil. Images were individually screened to exclude pathology. A continuous arterial spin-labelling technique [33] was used as previously described [75]. Interleaved images with and without labelling were acquired by using a gradient-echo echo-planar imaging (EPI) sequence (TR/TE: 4000/17, anterior-to-posterior phase encoding, flip angle 90°, bandwidth 3005 Hz/Pixel, field of view 22 cm). Image size was 64×64×15 voxels, slice thickness 6 mm with a gap of 1.5 mm, giving a voxel size of 3.44×3.44×7.50 mm. The inversion plane was located 8 cm below the central plane of the axial block of slices. A delay of 1 sec was inserted between the end of the labelling pulse (2 sec) and image acquisition to reduce transit artefacts. We made use of the SPM2 package (Wellcome Department of Cognitive Neurology, London; online at http://www.fil.ion.ucl.ac.uk) for realignment and stereotactic normalization to an EPI template (Montreal Neurological Institute, resampling size: 2×2×2 mm). Reconstruction of cerebral blood flow (CBF) values was obtained using software implementing equation (1) of ref. [33]. Volumes were smoothed with an isotropic Gaussian kernel of full width half maximum (FWHM) 8 mm.

### Statistical Analysis

The proportion of negative sentences, or the proportion of sentences formed overall, was entered in a generalized linear model (logistic regression) with age, sex, and OSPAN score as explanatory variables. The model was fit with the freely available statistical software R (version 2.9.2, The R Foundation for Statistical Computing, www.r-project.org, Vienna, Austria), which was also used for the tests of the previous section.

For the whole brain analysis, an explicit mask was obtained by thresholding the *a priori* thresholded tissue probability maps provided by the SPM package at 0.25 for gray or white matter. The cerebellar region and the lower brainstem were excluded manually in this mask because affected by large variances. All corrections in the Results section are voxel-level (peak-level, strong familywise error) corrections for the whole volume as defined by this mask. Cluster-level corrections are reported in the tables of the supplementary information available online. Significance values for voxel and cluster level inferences were computed based on the quantiles of the permutation distribution of the maximal *t* and of the largest cluster of contiguous suprathreshold voxels, respectively [76]. For cluster-level tests in the Supplementary Tables, clusters were defined *a priori* by the uncorrected threshold *p* = 0.005. In each test, 20 000 permutations were computed. Computations were carried out in MATLAB R2006b (The Mathworks, Natick, MA) installed on a machine equipped with a 64-bit Athlon processor (Advanced Micro Devices, Sunnyvale, CA) running Windows XP (Microsoft, Redmond, WA). For the generation of random numbers, the ‘MATLAB5 generator’ was used. Figures 1 and 2 were obtained by ‘fiducial mapping’ of the contrasts of interest on an average of 12 healthy individuals landmark- and surface-based atlas (PALS) using the freely available software package Caret [77], version 5.615 (http://brainvis.wustl.edu/wiki/index.php). The remaining figures are overlays of axial or sagittal slice of a template brain, generated with the freely available application MRIcroN [78], version beta 2 (http://www.cabiatl.com/mricro/mricron/index.html).

## Supporting Information

Table S1 Main contrast task vs. resting baseline. (DOC)

Table S2 Interaction valence×instruction. (DOC)

Table S3 Simple contrast emotional vs. neutral in spontaneous group. (DOC)

Table S4 Simple contrast emotional vs. neutral in instructed group. (DOC)

## References

[1] Beck AT (1976). Cognitive Theory and the Emotional Disorders.

[2] Taylor SE, Brown JD (1988). Illusion and well-being: A social psychological perspective on mental health.. Psychol Bull.

[3] Wenzlaff RM, Bates DE (1998). Unmasking a cognitive vulnerability to depression: How lapses in mental control reveal depressive thinking.. J Pers Soc Psychol.

[4] Wegner DM (1994). Ironic processes of mental control.. Psychol Rev.

[5] Ochsner KN, Gross JJ (2005). The cognitive control of emotion.. Trends Cogn Sci.

[6] Wegner DM, Erber R, Zanakos S (1993). Ironic processes in the mental control of mood and mood-related thought.. J Pers Soc Psychol.

[7] Wenzlaff RM, Wegner DM, Roper DW (1988). Depression and mental control: The resurgence of unwanted negative thoughts.. J Pers Soc Psychol.

[8] Phillips ML, Drevets WC, Rauch SL, Lane R (2003). Neurobiology of emotion perception I: The neural basis of normal emotion perception.. Biol Psychiatry.

[9] Phillips ML, Drevets WC, Rauch SL, Lane R (2003). Neurobiology of emotion perception II: Implications for major psychiatric disorders.. Biol Psychiatry.

[10] DeRubeis RJ, Siegle GJ, Hollon SD (2008). Cognitive therapy versus medication for depression: Treatment outcomes and neural mechanisms.. Nature Rev Neurosci.

[11] Thayer JF, Lane RD (2000). A model of neruovisceral integration in emotion regulation and dysregulation.. J Affect Dis.

[12] Egner T, Etkin A, Gale S, Hirsch J (2008). Dissociable neural systems resolve conflict from emotional versus nonemotional distracters.. Cereb Cortex.

[13] Phan KL, Fitzgerald DA, Nathan PJ, Moore GJ, Uhde TW (2005). Neural substrates for voluntary suppression of negative affect: A functional magnetic resonance imaging study.. Biol Psychiatry.

[14] Vuilleumier P, Armony JL, Driver J, Dolan JR (2001). Effects of attention and emotion of face processing in the human brain: An event-related fMRI study.. Neuron.

[15] Wager TD, Davidson ML, Hughes BL, Lindquist MA, Ochsner KN (2008). Prefrontal-subcortical pathways mediating successful emotion regulation.. Neuron.

[16] Damasio AR (1994). Descarte's Error: Emotion, Reason and the Human Brain.

[17] Slovic P, Finucane M, Peters E, MacGregor DG, Gilovich T, Griffin D, Kahneman D (2002). The affect heuristic.. Heuristics and Biases. The Psychology of Intuitive Judgment.

[18] Kahneman D, Tversky A (1979). Prospect theory: An analysis of decision under risk.. Econometrica.

[19] Tversky A, Kahneman D (1984). Extensional versus intuitive reasoning: The conjunction fallacy in probability judgment.. Psychol Rev.

[20] Bargh JA, Williams LE, Gross JJ (2007). The case for nonconscious emotion regulation.. Handbook of Emotion Regulation.

[21] Mauss IB, Bunge SA, Gross JJ (2007). Automatic emotion regulation.. Soc Pers Psychol Compass.

[22] Phillips ML, Ladouceur CD, Drevets WC (2008). A neural model of voluntary and automatic emotion regulation: Implications for understanding the pathophysiology and neurodevelopment of bipolar disorder.. Mol Psychiatry.

[23] Öhman A, Flykt A, Esteves F (2001). Emotion drives attention: Detecting the snake in the grass.. J Exp Psychol, Gen.

[24] Vuilleumier P (2005). How brains beware: Neural mechanisms of emotional attention.. Trends Cogn Sci.

[25] Rude SS, Valdey CR, Odom S, Ebrahimi A (2003). Negative cognitive biases predict subsequent depression.. Cogn Ther Res.

[26] Rude SS, Wenzlaff RM, Gibbs B, Vane J, Whitney T (2002). Negative processing bias predict subsequent depressive symptoms.. Cogn Emot.

[27] Barrett LF, Tugade MM, Engle RW (2004). Individual differences in working memory capacity and dual-process theories of mind.. Psychol Bull.

[28] Unsworth N, Heitz RP, Engle RW, Engle RW, Sedek G, Hecker U, McIntosh DN (2005). Working memory capacity in hot and cold cognition.. Cognitive Limitations in Aging and Psychopathology.

[29] Bargh JA, Ferguson MJ (2000). Beyond behaviourism: On the automaticity of higher mental processes.. Psychol Bull.

[30] Kahneman D, Frederick S, Gilovich T, Griffin D, Kahneman D (2002). Representativeness revisited: Attribute substitution in intuitive judgment.. Heuristics and Biases. The Psychology of Intuitive Judgment.

[31] Sloman SA (1996). The empirical case for two systems of reasoning.. Psychol Bull.

[32] Detre JA, Alsop DC, Moonen CTW, Bandettini PA (1999). Perfusion fMRI with arterial spin labelling.. Functional MRI.

[33] Wang J, Zhang Y, Wolf RL, Roc AC, Alsop DC (2005). Amplitude-modulated continuous arterial spin-labeling 3.0-T perfusion MR imaging with a single coil: Feasibility study.. Radiology.

[34] Braver TS, Cohen JD, Nystrom LE, Jonides J, Smith EE (1997). A parametric study of prefrontal cortex involvement in human working memory.. Neuro Image.

[35] Turner ML, Engle RW (1989). Is working memory capacity task dependent?. J Mem Lang.

[36] Baddeley AD (2007). Working Memory, Thought, and Action.

[37] Brewin CR, Beaton A (2002). Thought suppression, intelligence, and working memory capacity.. Behav Res Ther.

[38] De Neys W (2006). Automatic-heuristic and executive-analytic processing during reasoning: Chronometric and dual-task considerations.. Q J Exp Psychol.

[39] Stanovich KE, West RF (1998). Individual differences in rational thought.. J Exp Psychol, Gen.

[40] Binder JR, Desai RH, Graves WW, Conant LL (2009). Where is the semantic system? A critical review and meta-analysis of 120 functional neuroimaging studies.. Cereb Cortex.

[41] Shulman GL, Fiez JA, Corbetta M, Buckner RL, Miezin FM (1997). Common blood flow changes across visual tasks II. Decreases in cerebral cortex.. J Cogn Neurosci.

[42] Poldrack RA, Wagner AD, Prull MW, Desmond JE, Glover GH (1999). Functional specialization for semantic and phonological processing in the left inferior prefrontal cortex.. Neuro Image.

[43] Vigneau M, Beaucousin V, Hervé PY, Duffau H, Crivello F (2006). Meta-analyzing left hemisphere language areas: Phonology, semantics, and sentence processing.. Neuro Image.

[44] Paus T (1996). Location and function of the human frontal eye-fied: A selective review.. Neuropsychologia.

[45] Andreasen NC, O'Leary DS, Cizadlo T, Arnd S, Rezai K (1995). Remembering the past: Two facets of episodic memory explored with positron emission tomography.. Am J Psychiatry.

[46] Binder JR, Frost JA, Hammeke TA, Bellgowan PS, Rao SM (1999). Conceptual processing during the conscious resting state: A functional MRI study.. J Cogn Neurosci.

[47] Friston KJ, Frith CD, Liddle PF, Frackowiak RSJ (1991). Investigating a network model of word generation with positron emission tomography.. Proc R Soc Lond B.

[48] Mazoyer B, Zago L, Mellet E, Bricogne S, Etard O (2001). Cortical networks for working memory and executive functions sustain the conscious resting state in man.. Brain Res Bull.

[49] de Fockert JW, Rees G, Frith CD, Lavie N (2001). The role of working memory in visual selective attention.. Science.

[50] Kincade JM, Abrams RA, Astafiev SV, Shulman GL, Corbetta M (2005). An event-related functional magnetic resonance imaging study of voluntary and stimulus-driven orienting of attention.. J Neurosci.

[51] Grasby PM, Frith CD, Friston KJ, Simposon J, Fletcher PC (1994). A graded task approach to the functional mapping of brain areas implicated in auditory-verbal memory.. Brain.

[52] Raichle ME, MacLeod AM, Snyder AZ, Powers WJ, Gusnard DA (2001). A default mode of brain function.. Proc Natl Acad Sci USA.

[53] Bush G, Luu P, Posner MI (2000). Cognitive and emotional influences in anterior cingulate cortex.. Trends Cogn Sci.

[54] Kalisch R, Wiech K, Critchley HD, Dolan RJ (2006). Levels of appraisal: A medial prefrontal role in high-level appraisal of emotional material.. NeuroImage.

[55] Phelps EA, Delgado MR, Nearing KI, LeDoux JE (2004). Extinction learning in humans: Role of the amygdala and vmPFC.. Neuron.

[56] De Martino B, Kumaran D, Seymour B, Dolan RJ (2006). Frames, biases, and rational decision-making in the human brain.. Science.

[57] Sharot T, Riccardi AM, Raio CM, Phelps EA (2007). Neural mechanisms mediating optimism bias.. Nature.

[58] Buckner RL, Carroll DC (2007). Self-projection and the brain.. Trends Cogn Sci.

[59] Drevets WC (2000). Neuroimaging studies of mood disorder.. Biol Psychiatry.

[60] Mayberg HS, Liotti M, Brannan SK, McGinnis S, Mahurin RK (1999). Reciprocal limbic cortical function and negative mood. Converging PET findings in depression and normal sadness.. Am J Psychiatry.

[61] Corbetta M, Patel G, Shulman GL (2008). The reorienting system of the human brain: From environment to theory of mind.. Neuron.

[62] Serences JT, Shomstein S, Leber AB, Golay X, Egeth HE (2005). Coordination of voluntary and stimulus-driven attentional control in human cortex.. Psychol Sci.

[63] Rypma B, D'Esposito M (1999). The roles of prefrontal brain regions in components of working memory: Effects of memory load and individual differences.. Proc Natl Acad Sci USA.

[64] Badre D, D'Esposito M (2009). Is the rostro-caudal axis of the frontal lobe hierarchical?. Nature Rev Neurosci.

[65] Bar M (2007). The proactive brain: Using analogies and associations to generate predictions.. Trends Cogn Sci.

[66] Drevets WC, Raichle ME (1998). Reciprocal suppression of regional cerebral blood flow during emotional versus higher cognitive processes: Implications for interactions between emotion and cognition.. Cogn Emot.

[67] Simpson JR, Drevets WC, Snyder AZ, Gusnard DA, Raichle ME (2001). Emotion-induced changes in human medial prefrontal cortex: II. During anticipatory anxiety.. Proc Natl Acad Sci USA.

[68] Radloff LS (1977). The CES-D scale: A self-report depression scale for research in the general population.. Appl Psychol Meas.

[69] Hautzinger M, Bailer M (1993). Allgemeine Depressionsskala (ADS).

[70] Zeintl M, Kliegel M, Hofer SM (2007). The role of processing resources in age-related prospective and retrospective memory within old age.. Psychol Aging.

[71] Wenzlaff RM, Wegner DM, Pennebaker JW (1993). The mental control of depression: Psychological obstacles to emotional well-being.. Handbook of Mental Control.

[72] Aguirre GK, Detre JA, Zarahn E, Alsop DC (2002). Experimental design and the relative sensitivity of BOLD and perfusion fMRI.. Neuro Image.

[73] Bradley MM, Lang PJ (1994). Measuring emotion: The self-assessment manikin and the semantic differential.. J Behav Ther Exp Psychiatry.

[74] Pinheiro JC, Bates DM (2000). Mixed-Effects Models in S and S-PLUS.

[75] Viviani R, Sim EJ, Lo H, Richter S, Haffer S (2009). Components of variance in brain perfusion and the design of studies of individual differences: The baseline study.. Neuro Image.

[76] Holmes AP, Blair RC, Watson JDG, Ford I (1996). Nonparametric analysis of statistic images from functional mapping experiments.. J Cereb Blood Flow Metab.

[77] Van Essen DC (2005). A population-average, landmark- and surface-based (PASL) atlas of human cerebral cortex.. Neuro Image.

[78] Rorden C, Karnath HO, Bonilha L (2007). Improving lesion-symptom mapping.. J Cogn Neurosci.

