# Supplementary material for: The Neural Substrate of Positive Bias in Spontaneous Emotional Processing
Source: PLoS One. 2010 Nov 8;5(11):e15454. doi: 10.1371/journal.pone.0015454 (PMC2975711; doi:10.1371/journal.pone.0015454)
Supplement: Table S1 — Main contrast task vs. resting baseline. (DOC) [file pone.0015454.s001.doc]

# Supporting Table S1. Main contrast task vs. resting baseline

Activations

| Cl # | Brain area | Coord. (mm.) | *t* | *p* (uncorr.) | *p* (corr.) | *k* | *p* (cl.) |
| --- | --- | --- | --- | --- | --- | --- | --- |
| 1 | Occipital Inf L (BA18) | −36 −90 −8 | 11.065 | < 0.0001 | < 0.001 | 7339 | < 0.001 |
|  | Parietal Inf L (BA7) | −28 −60 44 | 7.596 | < 0.0001 | < 0.001 |  |  |
|  | Temp Mid L (BA21) | −48 −42 0 | 7.144 | < 0.0001 | < 0.001 |  |  |
|  | Occipital Mid L (BA18) | −32 −82 6 | 5.602 | < 0.0001 | 0.005 |  |  |
|  | Fusiform L (BA19) | −30 −70 −12 | 5.476 | < 0.0001 | 0.007 |  |  |
|  | Occipital Mid L (BA19) | −28 −78 22 | 5.298 | < 0.0001 | 0.014 |  |  |
|  | Temporal Mid L (BA22) | −56 −48 20 | 4.834 | < 0.0001 | 0.064 |  |  |
|  | Parietal Inf L (BA40) | −52 −48 44 | 4.565 | < 0.0001 | 0.134 |  |  |
|  | Fusiform L (BA37) | −38 −42 −20 | 2.945 | 0.0023 | 0.992 |  |  |
| 2 | Occipital Mid R (BA19) | 32 −76 26 | 6.663 | < 0.0001 | < 0.001 | 4385 | 0.001 |
|  | Occipital Inf R (BA18) | 34 −92 −6 | 6.298 | < 0.0001 | < 0.001 |  |  |
|  | SupraMarginal R (BA40) | 52 −40 46 | 6.128 | < 0.0001 | 0.001 |  |  |
|  | Occipital Mid R (BA19) | 32 −70 32 | 6.089 | < 0.0001 | 0.001 |  |  |
|  | Occipital Inf R (BA18) | 28 −98 0 | 5.906 | < 0.0001 | 0.002 |  |  |
|  | Occipital Sup R (BA7) | 32 −64 42 | 5.544 | < 0.0001 | 0.006 |  |  |
|  | Occipital Mid R (BA19) | 40 −88 12 | 5.376 | < 0.0001 | 0.010 |  |  |
|  | Angular R (BA40) | 34 −50 40 | 5.049 | < 0.0001 | 0.033 |  |  |
|  | Occipital Mid R (BA18) | 28 −86 14 | 4.101 | < 0.0001 | 0.375 |  |  |
|  | Lingual R (BA18) | 14 −76 −12 | 3.259 | 0.0009 | 0.939 |  |  |
|  | Fusiform R (BA37) | 34 −62 −10 | 2.791 | 0.0035 | 0.998 |  |  |
| 3 | Precentral L (BA6) | −44 0 44 | 10.845 | < 0.0001 | < 0.001 | 7499 | < 0.001 |
|  | Frontal Inf Tri L (BA48) | −52 18 6 | 9.899 | < 0.0001 | < 0.001 |  |  |
|  | Insula L (BA47) | −30 22 −2 | 9.632 | < 0.0001 | < 0.001 |  |  |
|  | Frontal Inf Tri L (BA44) | −54 24 32 | 5.362 | < 0.0001 | 0.011 |  |  |
|  | Pallidum L | −20 2 4 | 4.457 | < 0.0001 | 0.175 |  |  |
|  | Precentral L (BA4) | −40 −16 62 | 4.028 | 0.0001 | 0.429 |  |  |
|  | Frontal Inf Orb L (BA47) | −50 38 −10 | 3.845 | 0.0001 | 0.576 |  |  |
| 4 | Thalamus L | −10 −14 6 | 3.058 | 0.0017 | 0.980 | 56 | 0.838 |
| 5 | Front Inf Oper R (BA45) | 46 18 4 | 7.717 | < 0.0001 | < 0.001 | 1246 | 0.032 |
|  | Putamen R | 18 16 0 | 3.828 | 0.0002 | 0.589 |  |  |
| 6 | Temp Mid R (BA21) | 46 −32 −2 | 3.532 | 0.0004 | 0.811 | 49 | 0.868 |
| 7 | Thalamus R | 16 −14 10 | 3.214 | 0.0011 | 0.952 | 62 | 0.814 |
| 8 | Frontal Mid R (BA10) | 36 52 8 | 3.322 | 0.0008 | 0.917 | 41 | 0.900 |
| 9 | Supp Motor Area L (BA6) | −6 10 54 | 14.009 | < 0.0001 | < 0.001 | 6137 | < 0.001 |
|  | Cingulum Mid L (BA32) | −8 22 34 | 9.206 | < 0.0001 | < 0.001 |  |  |
|  | Precentral R (BA6) | 36 0 52 | 6.263 | < 0.0001 | < 0.001 |  |  |
|  | Precentral R (BA44) | 44 6 32 | 5.552 | < 0.0001 | 0.006 |  |  |
|  | Frontal Mid R (BA45) | 42 30 36 | 5.033 | < 0.0001 | 0.035 |  |  |
|  | Frontal Mid R (BA8) | 30 8 56 | 4.689 | < 0.0001 | 0.098 |  |  |
|  | Frontal Inf Tri R (BA44) | 54 24 30 | 4.027 | 0.0001 | 0.430 |  |  |
| 10 | Frontal Mid L (BA45) | −40 46 22 | 3.400 | 0.0006 | 0.885 | 90 | 0.703 |
| 11 | Angular R (BA41) | 48 −44 26 | 4.186 | < 0.0001 | 0.317 | 68 | 0.789 |
| 12 | Cingulum Mid L (BA23) | −4 −26 32 | 3.129 | 0.0014 | 0.969 | 26 | 0.953 |

Deactivations

| Cl # | Brain area | Coord. (mm.) | *t* | *p* (uncorr.) | *p* (corr.) | *k* | *p* (cl.) |
| --- | --- | --- | --- | --- | --- | --- | --- |
| 1 | Temporal Mid L (BA20) | −40 −18 −8 | −7.622 | < 0.0001 | < 0.001 | 4299 | 0.001 |
|  | Temporal Sup L (BA41) | −36 −34 14 | −5.924 | < 0.0001 | 0.002 |  |  |
|  | Temporal Sup L (BA42) | −58 −30 20 | −5.465 | < 0.0001 | 0.009 |  |  |
|  | Temp Pole Mid L (BA36) | −28 8 −38 | −3.754 | 0.0002 | 0.645 |  |  |
|  | Hippocampus L (BA37) | −34 −30 0 | −3.125 | 0.0014 | 0.970 |  |  |
| 2 | Temporal Sup R (BA48) | 52 −12 2 | −7.685 | < 0.0001 | < 0.001 | 7638 | < 0.001 |
|  | SupraMarginal R (BA48) | 60 −26 22 | −6.038 | < 0.0001 | 0.001 |  |  |
|  | ParaHippocamp R (BA28) | 22 2 −30 | −5.192 | < 0.0001 | 0.021 |  |  |
|  | Rolandic Oper R (BA48) | 56 −14 22 | −4.797 | < 0.0001 | 0.068 |  |  |
|  | ParaHippocamp R (BA36) | 28 −2 −28 | −4.709 | < 0.0001 | 0.088 |  |  |
|  | Temporal Mid R (BA21) | 56 0 −28 | −4.425 | < 0.0001 | 0.186 |  |  |
|  | Fusiform R (BA36) | 26 8 −44 | −4.058 | < 0.0001 | 0.406 |  |  |
|  | Cerebellum 4 5 R (BA30) | 26 −30 −24 | −3.042 | 0.0018 | 0.982 |  |  |
|  | SupraMarginal R (BA3) | 62 −20 44 | −2.947 | 0.0023 | 0.992 |  |  |
| 3 | ParaHipp L (BA36) | −20 −12 −30 | −3.573 | 0.0004 | 0.778 | 108 | 0.634 |
| 4 | Front Sup Med L (BA10) | −4 64 16 | −6.234 | < 0.0001 | 0.001 | 4096 | 0.001 |
|  | Rectus R (BA11) | 2 48 −18 | −6.020 | < 0.0001 | 0.001 |  |  |
|  | Frontal Sup L (BA10) | −16 64 16 | −5.317 | < 0.0001 | 0.014 |  |  |
|  | Front Mid Orb L (BA11) | −6 46 −12 | −4.594 | < 0.0001 | 0.121 |  |  |
|  | Cingulum Ant (BA25) | 0 30 6 | −3.984 | 0.0001 | 0.463 |  |  |
|  | Frontal Sup R (BA10) | 22 64 14 | −3.945 | 0.0001 | 0.493 |  |  |
| 5 | Fusiform L (BA37) | −28 −32 −24 | −3.382 | 0.0006 | 0.888 | 68 | 0.780 |
| 6 | Occipital Inf L (BA37) | −58 −62 −14 | −4.464 | < 0.0001 | 0.168 | 215 | 0.377 |
| 7 | Temporal Inf R (BA37) | 54 −62 −8 | −3.061 | 0.0017 | 0.979 | 31 | 0.933 |
| 8 | Subgenual Cing (BA25) | 2 4 −6 | −3.126 | 0.0014 | 0.970 | 62 | 0.805 |
| 9 | Frontal Inf Tri R (BA45) | 44 38 8 | −3.327 | 0.0008 | 0.913 | 23 | 0.960 |
| 10 | Precuneus L (BA23) | −12 −52 30 | −3.778 | 0.0002 | 0.625 | 103 | 0.650 |
| 11 | Precuneus R (BA5) | 6 −48 62 | −5.531 | < 0.0001 | 0.007 | 3379 | 0.003 |
|  | Parietal Sup L (BA2) | −22 −44 66 | −4.727 | < 0.0001 | 0.084 |  |  |
|  | Precuneus L (BA7) | 0 −78 50 | −4.195 | < 0.0001 | 0.314 |  |  |
|  | Precentral R (BA6) | 20 −22 72 | −4.017 | < 0.0001 | 0.437 |  |  |
|  | Paracentr Lobule L (BA4) | −10 −22 70 | −3.708 | 0.0002 | 0.683 |  |  |
|  | Parietal Sup R (BA7) | 26 −50 70 | −3.362 | 0.0007 | 0.897 |  |  |
|  | Postcentral R (BA4) | 24 −28 60 | −3.311 | 0.0008 | 0.919 |  |  |
|  | Paracentr Lobule R (BA4) | 4 −24 68 | −3.154 | 0.0013 | 0.963 |  |  |
|  | Cuneus L (BA18) | −4 −92 30 | −3.144 | 0.0013 | 0.965 |  |  |
| 12 | Occ Mid L (BA39) | −44 −78 32 | −3.876 | 0.0001 | 0.547 | 79 | 0.739 |
| 13 | Precentral R (BA4) | 44 −10 38 | −3.160 | 0.0012 | 0.962 | 44 | 0.881 |
| 14 | Cingulum Mid L (BA23) | −2 −4 42 | −3.923 | 0.0001 | 0.511 | 164 | 0.478 |
| 15 | Front Sup Med R (BA9) | 12 50 42 | −3.410 | 0.0006 | 0.874 | 52 | 0.847 |
| 16 | Frontal Mid L (BA8) | −24 24 46 | −4.163 | < 0.0001 | 0.334 | 278 | 0.296 |
| 17 | Cingulum Mid R (BA23) | 12 −22 44 | −3.234 | 0.0010 | 0.945 | 40 | 0.899 |
| 18 | Frontal Sup R (BA9) | 22 34 48 | −3.325 | 0.0008 | 0.914 | 69 | 0.776 |

Explanation of symbols: Cl #: cluster sequential number; BA: Brodmann Area; Coord. (mm): Montreal Neurological Institute Coordinates, in millimetres; *p* (uncorr.), significance level, uncorrected (df = 59); *p* (corr.): significance level, voxel-level correction; *p* (cl.): significance level, cluster-level correction; *k*: cluster extent (in voxels of size 2 × 2 × 2 mm). Clusters of at least 10 continguous voxels, with peaks reaching *p* = 0.005 at least 20 mm apart.
