# Supplementary material for: The Neural Substrate of Positive Bias in Spontaneous Emotional Processing
Source: PLoS One. 2010 Nov 8;5(11):e15454. doi: 10.1371/journal.pone.0015454 (PMC2975711; doi:10.1371/journal.pone.0015454)
Supplement: Table S2 — Interaction valence×instruction. (DOC) [file pone.0015454.s002.doc]

# Supporting Table S2. Interaction valence × instruction

Emotional vs. neutral larger in instructed than in spontaneous group

| Cl # | Brain area | Coord. (mm.) | *t* | *p* (uncorr.) | *p* (corr.) | *k* | *p* (cl.) |
| --- | --- | --- | --- | --- | --- | --- | --- |
| 1 | Fusiform R (BA20) | 42 −10 −34 | 3.21 | 0.0011 | 0.95 | 57 | 0.84 |
| 2 | Fusiform R (BA20) | 30 −30 −24 | 3.92 | 0.0001 | 0.52 | 81 | 0.74 |
| 3 | Fusiform L (BA37) | −34 −46 −22 | 4.07 | < 0.0001 | 0.40 | 66 | 0.80 |
| 4 | Fusiform L (BA37) | −34 −60 −18 | 3.81 | 0.0002 | 0.61 | 48 | 0.88 |
| 5 | Cerebellum 6 R (BA18) | 16 −60 −12 | 3.30 | 0.0008 | 0.93 | 43 | 0.90 |
| 6 | Occipital Sup L (BA17) | −10 −94 4 | 4.20 | < 0.0001 | 0.31 | 1853 | 0.01 |
|  | Occipital Mid L (BA39) | −40 −82 24 | 3.93 | 0.0001 | 0.51 |  |  |
|  | Parietal Sup L (BA7) | −22 −74 52 | 3.85 | 0.0001 | 0.58 |  |  |
|  | Parietal Inf L (BA7) | −36 −60 56 | 3.82 | 0.0002 | 0.60 |  |  |
|  | Occipital Sup L (BA18) | −12 −88 22 | 3.74 | 0.0002 | 0.66 |  |  |
|  | Lingual L (BA18) | −10 −80 −2 | 3.66 | 0.0003 | 0.73 |  |  |
|  | Occipital Mid L (BA18) | −28 −94 12 | 3.41 | 0.0006 | 0.88 |  |  |
|  | Parietal Inf L (BA7) | −30 −76 44 | 3.39 | 0.0006 | 0.89 |  |  |
|  | Cuneus L (BA18) | 2 −76 22 | 3.23 | 0.0010 | 0.95 |  |  |
|  | Occipital Sup L (BA18) | −18 −82 34 | 2.91 | 0.0026 | 0.99 |  |  |
|  | Parietal Sup L (BA7) | −30 −50 58 | 2.84 | 0.0031 | 1.00 |  |  |
| 7 | Occipital Mid R (BA18) | 38 −84 8 | 3.62 | 0.0003 | 0.76 | 186 | 0.44 |
| 8 | Calcarine R (BA17) | 12 −96 2 | 3.12 | 0.0014 | 0.97 | 27 | 0.95 |
| 9 | Putamen L (BA48) | −26 14 6 | 3.26 | 0.0009 | 0.94 | 233 | 0.36 |
| 10 | Putamen R (BA48) | 28 −10 12 | 3.41 | 0.0005 | 0.88 | 132 | 0.57 |
| 11 | Frontal Inf Oper L (BA48) | −48 6 8 | 3.35 | 0.0006 | 0.91 | 51 | 0.87 |
| 12 | Postcentral L (BA4) | −40 −8 34 | 3.09 | 0.0015 | 0.98 | 30 | 0.94 |
| 13 | Frontal Mid L (BA45) | −44 32 32 | 3.08 | 0.0016 | 0.98 | 20 | 0.97 |
| 14 | Precentral R (BA6) | 44 2 52 | 5.13 | < 0.0001 | 0.02 | 416 | 0.19 |
|  | Frontal Mid R (BA8) | 30 18 46 | 2.73 | 0.0042 | 1.00 |  |  |
| 15 | Precentral L (BA6) | −34 −4 54 | 4.36 | < 0.0001 | 0.21 | 450 | 0.17 |
| 16 | Postcentral L (BA2) | −46 −32 52 | 3.19 | 0.0012 | 0.96 | 183 | 0.44 |
| 17 | Postcentral R (BA4) | 42 −26 60 | 2.85 | 0.0030 | 1.00 | 24 | 0.96 |
| 18 | Frontal Sup L (BA8) | −16 10 58 | 2.99 | 0.0021 | 0.99 | 17 | 0.98 |
| 19 | Supp Motor Area R (BA8) | 10 26 56 | 3.20 | 0.0011 | 0.96 | 14 | 0.98 |
| 20 | Parietal Sup R (BA40) | 40 −50 64 | 3.84 | 0.0002 | 0.58 | 33 | 0.93 |

Emotional vs. neutral larger in spontaneous than in instructed group

| Cl # | Brain area | Coord. (mm.) | *t* | *p* (uncorr.) | *p* (corr.) | *k* | *p* (cl.) |
| --- | --- | --- | --- | --- | --- | --- | --- |
| 1 | Temporal Inf L (BA20) | −50 −24 −22 | −2.87 | 0.0029 | 1.00 | 12 | 0.99 |
| 2 | Frontal Inf Orb R (BA38) | 32 20 −22 | −3.16 | 0.0013 | 0.97 | 14 | 0.98 |
| 3 | Temporal Inf L (BA37) | −60 −60 −20 | −3.09 | 0.0015 | 0.98 | 12 | 0.99 |
| 4 | Hypothalamus (BA25) | 4 4 −16 | −2.99 | 0.0021 | 0.99 | 10 | 0.99 |
| 5 | Calc L (BA18) | 4 −95 −16 | −2.87 | 0.0028 | 1.00 | 19 | 0.97 |
| 6 | Occ Inf R (BA18) | 40 −94 −4 | −3.35 | 0.0007 | 0.90 | 12 | 0.99 |
| 7 | Ant Cing L (BA11) | −8 38 6 | −3.01 | 0.0019 | 0.99 | 14 | 0.98 |
| 8 | Front Sup Med L (BA10) | −2 64 30 | −2.99 | 0.0020 | 0.99 | 20 | 0.97 |
| 9 | SupraMarginal L (BA2) | −64 −20 30 | −3.19 | 0.0011 | 0.96 | 36 | 0.92 |

Explanation of symbols: Cl #: cluster sequential number; BA: Brodmann Area; Coord. (mm): Montreal Neurological Institute Coordinates, in millimetres; *p* (uncorr.), significance level, uncorrected (df = 59); *p* (corr.): significance level, voxel-level correction; *p* (cl.): significance level, cluster-level correction; *k*: cluster extent (in voxels of size 2 × 2 × 2 mm). Clusters of at least 10 continguous voxels, with peaks reaching *p* = 0.005 at least 20 mm apart.
