# Supplementary material for: The Neural Substrate of Positive Bias in Spontaneous Emotional Processing
Source: PLoS One. 2010 Nov 8;5(11):e15454. doi: 10.1371/journal.pone.0015454 (PMC2975711; doi:10.1371/journal.pone.0015454)
Supplement: Table S3 — Simple contrast emotional vs. neutral in spontaneous group. (DOC) [file pone.0015454.s003.doc]

# Supporting Table S3. Simple contrast emotional vs. neutral in spontaneous group

Emotional larger than neutral

| Cl # | Brain area | Coord. (mm.) | *t* | *p* (uncorr.) | *p* (corr.) | *k* | *p* (cl.) |
| --- | --- | --- | --- | --- | --- | --- | --- |
| 1 | Hypothal./Hypophysis | 12 4 −28 | 3.17 | 0.0018 | 0.98 | 29 | 0.92 |
| 2 | Hypothal./Hypophysis | −6 8 −28 | 3.73 | 0.0004 | 0.80 | 10 | 0.99 |
| 3 | Temporal Inf L (BA20) | −54 −22 −26 | 3.08 | 0.0023 | 0.99 | 16 | 0.97 |
| 4 | Calc L (BA17) | 2 −98 −14 | 3.12 | 0.0020 | 0.99 | 15 | 0.97 |
| 5 | Insula R (BA48) | 40 −14 −4 | 2.99 | 0.0029 | 0.99 | 14 | 0.98 |
| 6 | Cigulum Ant L (BA11) | −8 38 6 | 3.20 | 0.0017 | 0.98 | 43 | 0.86 |
| 7 | Cingulum Ant (BA24) | 0 26 22 | 3.14 | 0.0019 | 0.98 | 69 | 0.75 |
| 8 | Rolandic Oper L (BA48) | −40 −24 24 | 4.81 | < 0.0001 | 0.18 | 131 | 0.53 |
| 9 | Frontal Inf Tri L (BA45) | −56 28 22 | 3.08 | 0.0022 | 0.99 | 12 | 0.98 |
| 10 | Postcentral L (BA43) | −64 −10 28 | 4.72 | < 0.0001 | 0.22 | 85 | 0.68 |
| 11 | Front Sup Med L (BA10) | −6 58 26 | 2.97 | 0.0030 | 0.99 | 18 | 0.97 |

Neutral larger than emotional

| Cl # | Brain area | Coord. (mm.) | *t* | *p* (uncorr.) | *p* (corr.) | *k* | *p* (cl.) |
| --- | --- | --- | --- | --- | --- | --- | --- |
| 1 | Fusiform R (BA20) | 30 −30 −24 | −3.54 | 0.0007 | 0.89 | 26 | 0.94 |
| 2 | Fusiform L (BA37) | −30 −46 −8 | −3.38 | 0.0011 | 0.94 | 58 | 0.80 |
| 3 | Fusiform L (BA37) | −36 −60 −16 | −3.36 | 0.0011 | 0.95 | 31 | 0.92 |
| 4 | Frontal Sup Orb L (BA11) | −18 52 −8 | −3.29 | 0.0013 | 0.96 | 18 | 0.97 |
| 5 | Front Sup Med R (BA10) | 16 58 0 | −3.54 | 0.0007 | 0.89 | 26 | 0.94 |
| 6 | Frontal Inf Tri L (BA45) | −44 44 2 | −3.33 | 0.0012 | 0.95 | 23 | 0.95 |
| 7 | Putamen R (BA48) | 32 −8 8 | −3.30 | 0.0013 | 0.96 | 62 | 0.78 |
| 8 | Frontal Inf Tri L (BA45) | −36 36 6 | −3.37 | 0.0011 | 0.94 | 40 | 0.88 |
| 9 | Occipital Mid L (BA19) | −40 −86 22 | −3.16 | 0.0018 | 0.98 | 55 | 0.81 |
| 10 | Occipital Sup L (BA18) | −16 −88 24 | −3.43 | 0.0009 | 0.93 | 41 | 0.87 |
| 11 | Cuneus R (BA18) | 6 −82 28 | −3.32 | 0.0012 | 0.96 | 55 | 0.81 |
| 12 | Occipital Mid R (BA19) | 38 −72 38 | −3.87 | 0.0003 | 0.72 | 102 | 0.63 |
| 13 | Parietal Sup L (BA7) | −30 −50 60 | −3.95 | 0.0002 | 0.67 | 590 | 0.11 |
|  | Occipital Mid L (BA7) | −34 −62 36 | −3.53 | 0.0007 | 0.89 |  |  |
|  | Parietal Inf L (BA40) | −48 −48 48 | −3.46 | 0.0009 | 0.92 |  |  |
|  | Occipital Sup L (BA7) | −24 −68 36 | −3.35 | 0.0011 | 0.95 |  |  |
| 14 | Occipital Sup R (BA7) | 24 −66 38 | −3.31 | 0.0013 | 0.96 | 43 | 0.87 |
| 15 | Postcentral R (BA3) | 48 −22 40 | −3.71 | 0.0004 | 0.82 | 92 | 0.66 |
| 16 | Precentral L (BA6) | −36 −2 54 | −4.55 | < 0.0001 | 0.30 | 827 | 0.07 |
|  | Precentral L (BA6) | −46 −6 48 | −3.13 | 0.0020 | 0.98 |  |  |
| 17 | Precentral R (BA6) | 44 4 52 | −5.67 | < 0.0001 | 0.03 | 493 | 0.14 |
|  | Frontal Mid R (BA46) | 32 20 46 | −3.26 | 0.0014 | 0.97 |  |  |
| 18 | Postcentral L (BA3) | −48 −22 52 | −3.22 | 0.0016 | 0.97 | 17 | 0.97 |
| 19 | Postcentral R (BA4) | 40 −26 60 | −4.07 | 0.0002 | 0.59 | 150 | 0.49 |
| 20 | Parietal Sup R (BA40) | 40 −48 64 | −3.84 | 0.0003 | 0.74 | 36 | 0.90 |

Explanation of symbols: Cl #: cluster sequential number; BA: Brodmann Area; Coord. (mm): Montreal Neurological Institute Coordinates, in millimetres; *p* (uncorr.), significance level, uncorrected (df = 29); *p* (corr.): significance level, voxel-level correction; *p* (cl.): significance level, cluster-level correction; *k*: cluster extent (in voxels of size 2 × 2 × 2 mm). Clusters of at least 10 continguous voxels, with peaks reaching *p* = 0.005 at least 20 mm apart.
