# Supplementary material for: The Neural Substrate of Positive Bias in Spontaneous Emotional Processing
Source: PLoS One. 2010 Nov 8;5(11):e15454. doi: 10.1371/journal.pone.0015454 (PMC2975711; doi:10.1371/journal.pone.0015454)
Supplement: Table S4 — Simple contrast emotional vs. neutral in instructed group. (DOC) [file pone.0015454.s004.doc]

# Supporting Table S4. Simple contrast emotional vs. neutral in instructed group

Emotional larger than neutral

| Cl # | Brain area | Coord. (mm.) | *t* | *p* (uncorr.) | *p* (corr.) | *k* | *p* (cl.) |
| --- | --- | --- | --- | --- | --- | --- | --- |
| 1 | Temporal Inf R (BA20) | 44 −10 −38 | 2.96 | 0.0031 | 1.00 | 11 | 1.00 |
| 2 | Fusiform L (BA37) | −34 −46 −22 | 3.56 | 0.0006 | 0.94 | 14 | 0.99 |
| 3 | Temp Pole Sup R (BA38) | 46 26 −18 | 3.70 | 0.0005 | 0.90 | 39 | 0.94 |
| 4 | Calcarine L (BA17) | −12 −86 6 | 6.65 | < 0.0001 | < 0.01 | 1179 | 0.02 |
|  | Occipital Mid L (BA19) | −34 −82 16 | 2.98 | 0.0029 | 1.00 |  |  |
| 5 | Periacq gray | 2 −32 −4 | 3.49 | 0.0008 | 0.96 | 88 | 0.73 |
| 9 | Lingual R (BA18) | 16 −58 −10 | 4.13 | 0.0001 | 0.65 | 137 | 0.55 |
| 6 | Hippocampus R (BA20) | 26 −18 −8 | 3.13 | 0.0020 | 1.00 | 12 | 1.00 |
| 7 | Putamen L | −24 16 −8 | 3.29 | 0.0013 | 0.99 | 26 | 0.97 |
| 8 | Frontal Mid R (BA10) | 38 60 4 | 3.38 | 0.0010 | 0.98 | 62 | 0.85 |
| 9 | Temporal Sup R (BA48) | 64 −4 6 | 3.40 | 0.0010 | 0.98 | 58 | 0.86 |
| 10 | Calcarine R (BA18) | 14 −98 4 | 3.09 | 0.0022 | 1.00 | 15 | 0.99 |
| 11 | Temporal Mid R (BA22) | 64 −36 8 | 4.11 | 0.0001 | 0.66 | 265 | 0.28 |
| 12 | Rolandic Oper L (BA48) | −46 6 10 | 4.47 | < 0.0001 | 0.42 | 582 | 0.09 |
|  | Rolandic Oper L (BA48) | −42 −26 20 | 3.49 | 0.0008 | 0.96 |  |  |
| 13 | Temporal Mid R (BA21) | 64 −52 10 | 3.68 | 0.0005 | 0.91 | 44 | 0.92 |
| 14 | Temporal Mid R (BA37) | 44 −64 14 | 3.62 | 0.0006 | 0.93 | 133 | 0.56 |
| 15 | Temporal Sup L (BA22) | −62 −26 12 | 3.18 | 0.0018 | 1.00 | 22 | 0.98 |
| 16 | Heschl R (BA48) | 36 −22 12 | 3.13 | 0.0020 | 1.00 | 15 | 0.99 |
| 17 | Caudate L | −12 6 10 | 2.97 | 0.0030 | 1.00 | 11 | 1.00 |
| 18 | Temporal Mid L (BA21) | −50 −50 14 | 3.14 | 0.0020 | 1.00 | 22 | 0.98 |
| 19 | Precuneus R (BA30) | 12 −50 18 | 3.23 | 0.0015 | 0.99 | 51 | 0.89 |
| 20 | Insula R (BA48) | 32 0 18 | 3.18 | 0.0017 | 1.00 | 10 | 1.00 |
| 21 | Precuneus L (BA23) | −10 −54 20 | 3.09 | 0.0022 | 1.00 | 42 | 0.93 |
| 22 | Cingulum Ant L (BA24) | −4 18 32 | 3.34 | 0.0012 | 0.98 | 42 | 0.93 |
| 23 | Frontal Mid L (BA45) | −46 32 30 | 3.62 | 0.0005 | 0.93 | 42 | 0.93 |
| 24 | Parietal Sup L (BA7) | −20 −76 52 | 4.19 | 0.0001 | 0.61 | 179 | 0.44 |
|  | Occipital Mid L (BA19) | −36 −82 40 | 3.23 | 0.0016 | 0.99 |  |  |

Neutral larger than emotional

| Cl # | Brain area | Coord. (mm.) | *t* | *p* (uncorr.) | *p* (corr.) | *k* | *p* (cl.) |
| --- | --- | --- | --- | --- | --- | --- | --- |
| 1 | Temporal Inf L (BA20) | −62 −34 −20 | −3.42 | 0.0009 | 0.97 | 20 | 0.99 |
| 2 | Rectus L (BA11) | −6 50 −16 | −3.15 | 0.0019 | 1.00 | 10 | 1.00 |
| 3 | Temporal Inf R (BA20) | 54 −42 −12 | −3.24 | 0.0015 | 0.99 | 10 | 1.00 |
| 4 | Frontal Inf Orb L (BA47) | −50 36 −10 | −3.51 | 0.0007 | 0.96 | 15 | 0.99 |
| 5 | Frontal Mid L (BA46) | −36 50 12 | −3.65 | 0.0005 | 0.92 | 63 | 0.84 |
| 6 | Frontal Sup R (BA8) | 20 14 62 | −3.65 | 0.0005 | 0.92 | 32 | 0.96 |

Explanation of symbols: Cl #: cluster sequential number; BA: Brodmann Area; Coord. (mm): Montreal Neurological Institute Coordinates, in millimetres; *p* (uncorr.), significance level, uncorrected (df = 29); *p* (corr.): significance level, voxel-level correction; *p* (cl.): significance level, cluster-level correction; *k*: cluster extent (in voxels of size 2 × 2 × 2 mm). Clusters of at least 10 continguous voxels, with peaks reaching *p* = 0.005 at least 20 mm apart.
